# Factors associated with breast cancer screening intention in Kathmandu Valley, Nepal

**DOI:** 10.1371/journal.pone.0245856

**Published:** 2021-01-22

**Authors:** Divya Bhandari, Akira Shibanuma, Junko Kiriya, Suzita Hirachan, Ken Ing Cherng Ong, Masamine Jimba

**Affiliations:** 1 Department of Community and Global Health, Graduate School of Medicine, The University of Tokyo, Bunkyo, Japan; 2 Tribhuvan University Teaching Hospital, Kathmandu, Nepal; Qazvin University of Medical Sciences, ISLAMIC REPUBLIC OF IRAN

## Abstract

**Background:**

Breast cancer burden is increasing in low-income countries (LICs). Increasing incidence and delayed presentation of breast cancer are mainly responsible for this burden. Many women do not participate in breast cancer screening despite its effectiveness. Moreover, studies are limited on the barriers associated with low utilization of breast cancer screening in LICs. This study identified breast cancer screening behavior and factors associated with breast cancer screening intention among women in Kathmandu Valley, Nepal.

**Methods:**

A cross-sectional study was conducted among 500 women living in five municipalities of Kathmandu Valley, Nepal. Data were collected from July to September 2018, using a structured questionnaire. Interviews were conducted among women selected through proportionate random household sampling. This study was conceptualized using the theory of planned behavior, fatalism, perceived susceptibility, and perceived severity. The outcome variables included: the intention to have mammography (MMG) biennially, the intention to have clinical breast examination (CBE) annually, and the intention to perform breast self-examination (BSE) monthly. Analysis was conducted separately for each outcome variable using partial proportional odds model.

**Results:**

Out of 500 women, 3.4% had undergone MMG biennially, 7.2% CBE annually, and 14.4% BSE monthly. Women with a positive attitude, high subjective norms, and high perceived behavioral control were more likely to have the intention to undergo all three screening methods. Similarly, women were more likely to have intention to undergo CBE and MMG when they perceived themselves susceptible to breast cancer. Conversely, women were less likely to have intention to undergo CBE when they had high fatalistic beliefs towards breast cancer.

**Conclusion:**

Women in this study had poor screening behavior. The practice of breast self-examination was comparatively higher than clinical breast examination and mammography. Multidimensional culturally sensitive interventions are needed to enhance screening intentions. Efforts should be directed to improve attitude, family support, and fatalistic belief towards cancer. Furthermore, the proper availability of screening methods should be ensured while encouraging women to screen before the appearance of symptoms.

## Introduction

Breast cancer is a leading global health problem. There were 2.1 million cases diagnosed in 2018, and 627,000 died of breast cancer globally [1, 2]. Among women, it is the most commonly diagnosed cancer and the leading cause of cancer deaths. The mortality to incidence ratio of breast cancer in low-income countries (LICs) is three times higher than in high-income countries [2]. This highlights the increasing burden of breast cancer in LICs. Moreover, the incidence of breast cancer is increasing in LICs due to physical inactivity, changes in reproductive patterns, and unhealthy dietary habits [3, 4].

Breast cancer screening is an effective prevention strategy to reduce breast cancer burden [5]. Mammography (MMG), clinical breast examination (CBE), and breast self-examination (BSE) are three widely practiced screening tests. MMG is recommended as a standard screening test globally [5]. However, considering the cost-effectiveness, CBE, and BSE are also recommended for low-resource settings [6–8]. Early detection of breast cancer saves lives, preserves the quality of life, and prevents catastrophic out-of-pocket payments [9, 10]. In addition, early diagnosed cases can be successfully treated with less extensive breast conservative surgery [11].

However, the late presentation of breast cancer is still common, and it has worsened the economic and health conditions of LICs such as Nepal [12, 13]. Majority of women in LICs tend to seek medical treatment in the late stages of cancer [14]. According to a clinical study conducted in Nepal, most breast cancer patients sought treatment at late stages (stages II and III) with an average tumor size of two to five centimeters [15]. This delay has led to increased tumor size, complicated treatment, and finally, premature mortality in Nepal [16–18]. Further, the absence of universal health coverage adds a substantial financial burden to the patients and their families [19, 20].

Considering these detrimental consequences of breast cancer, it is necessary to identify factors associated with low screening intentions. So far, different reasons have been identified for low screening intentions, such as lack of education, absence of family history, poor access to screening, financial difficulties, and fear [21, 22]. However, a great part of variance remained unexplained due to the inclusion of limited factors in those studies [23]. This study builds on previous research by including relevant factors based on the concept of the theory of planned behavior (TPB), perceived susceptibility, perceived severity, and fatalism [23].

Furthermore, studies on breast cancer screening intention and behavior are limited in LICs. Therefore, this study aimed 1) to examine breast cancer screening behavior among women in Kathmandu Valley, Nepal, and 2) to identify the factors associated with the intentions to perform breast cancer screening tests (MMG, CBE, and BSE).

## Methods

### Settings and participants

A cross-sectional study was conducted among Nepalese women residing in Kathmandu Valley, Nepal. Proportional random sampling was used to recruit the participants in this study. Women were selected from five municipalities (Kathmandu, Bhaktapur, Lalitpur, Kirtipur, and Madhyapur Thimi) which are considered as a core urban area of the Valley [24]. The study was focused on these areas because the urban population is at higher risk of breast cancer, and screening services (especially mammography) are available in those areas compared to other areas of Nepal. From five municipalities, a total of 20 wards were selected randomly. A random household sampling was conducted proportionately to the number of households in those wards.

This study recruited women aged 40 years and above as eligible participants considering the age of women recommended to undergo mammography by the American Cancer Society [5]. Women with a history of breast cancer were excluded from the study.

The sample size was determined using the OpenEpi sample size calculator for a cross-sectional study with a power of 80%, and a confidence level of 95%. As reported in a previous study with a similar study design [25], our calculation was based on the assumption that 7.1% of people with good literacy and 1.8% of people with low literacy practiced breast cancer screening. The calculated minimum sample size was 476. Considering 10% dropout, the target sample size was increased to 529. Compare to other factors included in this study, literacy required a larger sample size to show association with the independent variable, so it was considered for calculating the sample size.

### Survey tools

Validated questionnaires from previous studies were used in this study [26–29]. Formal permissions were received from authors before using their questionnaire. The questionnaire unavailable in the Nepalese language was translated, back-translated, and further reviewed by experts. The expert panel included two breast cancer experts, two Nepali language experts, two public health experts, and three local leaders. It was then pretested among 50 women (around 10% of the target sample size) living in ward number 32 of Kathmandu district. The data collected from the pretest were not included in the main analysis. Inter-item reliability coefficients (Cronbach’s alpha) were checked to ensure the internal consistency of items. The Cronbach’s alpha for each construct included in the questionnaire was above 0.7, which is mentioned below. Some of the wordings were rephrased to make it understandable among local women.

### Exposure variables and assessment

This study included perceived susceptibility, perceived severity, and fatalism along with the constructs of the TPB (attitude, subjective norms, and perceived behavioral control) as exposure variables.

#### Attitude, subjective norms, and perceived behavioral control (constructs of the TPB)

TPB is a health behavioral model, which presumes that attitudes towards behavior, subjective norms, and, perceived behavioral control, together determine the individual intention and behavior [30]. The questionnaire developed by Gaston Godin was used in this research to measure the attitudes towards behavior, subjective norms, and perceived behavioral control [26]. Each construct (attitude, subjective norms, and perceived behavioral control) consists of three items that were measured on a five-point Likert scale (1: strongly disagree/strongly oppose/very difficult/very improbable to 5:strongly agree/ strongly favor/very easy/very probable). All three items were summed up to calculate the total scores (range 3 to15) for each construct. A higher score indicated positive/good attitudes, higher subjective norms, and higher perceived behavior, respectively. The Cronbach’s alpha for this scale ranged from 0.79 to 0.91 in this study.

#### Fatalism

Fatalism is a psychological doctrine where individuals believe all events are fated, and human beings cannot change or control the outcomes [31, 32]. It was measured using the revised Powe fatalism inventory scale initially developed by Barbara Powe and later modified by Rachel Mayo [27]. The revised Fatalism scale consists of a total of 11 items (yes/no) grouped in four subscales: predetermination (five items), religiosity (one item), inevitable death (two items), and pessimism (three items). The total score for fatalism (range 0–11) was calculated by adding responses of all 11 items which were coded as yes = 1 and no = 0. Fatalism was treated as a continuous variable and its’ higher value represented higher fatalism. The Cronbach’s alpha for the fatalism scale was 0.88 in this study.

#### Perceived susceptibility and severity

Perceived susceptibility is the degree to which a person considers themselves susceptible to disease [33], and perceived severity is perception regarding the consequences of disease [34]. They were measured using the validated ‘Nepalese Health Belief Model scale’ [28]. It consists of five items to measure perceived susceptibility and seven items to measure perceived severity. Responses were rated on a five-point Likert scale (1 ‘strongly disagree’ to 5 ‘strongly agree’). The total score for susceptibility was calculated by adding the scores of all five items. Likewise, the total score for severity was calculated by adding all seven items. A higher score meant higher susceptibility and higher severity. The Cronbach’s alpha for susceptibility subscale was 0.97, and the severity subscale was 0.87 in this study.

### Outcome variable and assessment

#### Breast cancer screening behavior

The main outcome variable for the first objective of this study was breast cancer screening behavior. We asked participants whether they had undergone each screening method (MMG, CBE, and BSE) and assessed their screening behavior as a binary variable (yes/no). For those who answered yes, further follow-up question on frequency of screening was also asked.

#### Intention to have breast cancer screening

The main outcome variable for the second objective of this study was the intention to undergo each breast cancer screening methods. Participants reported their intentions to undergo MMG biennially, CBE annually, and BSE monthly on a five-point Likert scale (1 ‘strongly disagree’ to 5 ‘strongly agree’) each.

### Potential confounders and assessment

The following variables were also assessed as potential confounders.

#### Knowledge of breast cancer

Participants with higher knowledge of breast cancer are more likely to have a higher intention or probability of undergoing screening [34]. At the same time, knowledge can bring positive change in attitude and the fatalistic belief of the person [35]. Knowledge was measured using 21-item ‘Modified Comprehensive Breast Cancer Knowledge Test’ (yes/no/don’t know) questionnaire, previously validated in the Nepalese context [29]. The correct response was scored ‘1’ while incorrect answer or ‘don’t know’ responses were scored ‘0’. The total score for knowledge was calculated by adding scores of all 21 items and it was treated as a continuous variable. The Cronbach’s alpha for this scale was 0.83 in this study.

#### Socio-demographic characteristics

This study adopted socio-demographic variables from the Nepal Demographic Health Survey 2016 [36]. The variables included were age, educational level, religion, ethnicity, current occupation, husband’s education, husband’s occupation, family income and time to the nearest screening facility. Age is considered as one of the factors to influence screening behavior and intention in the previous study. Also, with an increase in age, people are more likely to develop fatalistic beliefs towards cancer and might perceive themselves less susceptible to cancer [36, 37]. Other factors like education, income was associated with the screening behavior and intention in a previous study [38]. These factors are also more likely to influence perceived behavioral control and attitude towards screening [38]. Additionally, the family history of breast cancer, participation in an awareness program on breast cancer, and any family member from the health field was also asked (yes/no). Family history and family member from the health field is also likely to influence both attitude and behavior [39].

### Data collection

Data were collected through face-to-face interviews using a structured questionnaire from July to September 2018. Data collection was done by the principal researcher along with three trained research assistants with a public health background. Data collection in the Lalitpur municipality was also assisted by medical doctors from KIST medical college located in that municipality. The 30-minute long interview was conducted by visiting each household.

### Data analysis

Data were entered in Epi Data version 3.1 and exported to Stata version 13.1 for analyses. Data were analyzed using the partial proportional odds model (PPOM). Due to the ordinal nature of outcome variables, data were first fitted with the standard ordinal logistic regression (i.e. proportional odds model (POM)) model. However, POM was found inappropriate due to the violation of a proportional odds assumption for some independent variables. The proportional odds assumption was checked using a series of Wald tests, and Brant tests [40, 41]. The other alternatives were PPOM and fully unconstrained generalized ordered logit model (GOLM). PPOM relaxes the proportional odds assumptions for only those variables where it is violated. Whereas, GOLM relaxes the assumption for all variables, even if the assumption was violated by a few of them [42], resulting in too many parameters. Therefore, PPOM is usually considered a more efficient alternative to the GOLM [42]. Nonetheless, both PPOM and GOLM models were compared based on the Akaike Information Criterion (AIC) and Bayes’ Information Criterion (BIC) [43], and the PPOM model was found to have smaller AIC and BIC statistics. We, therefore, used PPOM as the final model to assess the factors associated with the intention to undergo screening.

This study had three outcome variables (Intention to undergo MMG, CBE, and BSE) measured in 5 categories (1: ‘Strongly Disagree’ (SD), 2: ‘Disagree’(D), 3: ‘Neutral’ (N), 4: ‘Agree’ (A), and 5: ‘Strongly Agree’ (SA). Therefore, a total of three separate PPOMs were fitted and analyzed. For the MMG model, all variables fulfilled the proportional assumption. However, in the case of the CBE model, the assumption was violated for the ‘perceived behavioral control’ variable; and for the BSE model, the assumption was violated for ‘knowledge of breast cancer’, ‘attitude’, ‘subjective norms’, and ‘perceived behavioral control’. In PPOM, for variables that meet the proportional odds assumption, only one odd ratio is reported; and for variables that fail to meet the assumption, multiple odd ratios are reported. As a result, Table 5 presents multiple adjusted odd ratios(AOR) for the variables that violate the assumption which has been labeled and also noted in the footnote of the table using symbols ’a’, ’b’, and ’c’ where, a = AOR for SD&D versus N, A and SA; b = AOR for SD&D or N versus A and SA; c = AOR for SD&D or N or A versus SA. Although the responses of outcome variables were measured on 5-level categories, responses in the SD category were found to be limited i.e. less than 15 for all three outcomes (as evident from Table 4). In particular, cross-tabulation showed that there were no respondents for the SD category corresponding to some categories of included independent variables. Therefore, “SD” category was merged with “D” category (renamed as “SD&D” category) to reduce false precision and to improve the stability and generalizability of the results.

Furthermore, before running the multivariable analysis, the multicollinearity test between independent variables was checked using the Variance Inflation Factor (VIF). Variable having a VIF of 10 or higher were excluded from the analyses [44]. The maximum VIF was 3.5 among the included variables in the final models. Table 5 present the result of the final multivariable PPOM models with AOR. Adjusted variables include age, no of children, education, occupation, family income, family members from a health background, the time required to reach a nearby health facility, and family history of breast cancer. Statistical significance for the final model was set at p<0.05.

### Ethics

This study obtained approval from the Research Ethics Committee of the Graduate School of Medicine, the University of Tokyo, Japan (SN 12034), and also from the Ethical Review Board of Nepal Health Research Council (SN 339/2018). The site approval letter was obtained from concerned district public health offices and metropolitan offices. Before data collection, informed written consent was obtained from the women participating in the study. Women participated voluntarily and their identity was kept anonymous by using identification codes.

## Results

Out of 529 women who were approached for this study, 500 agreed to participate. There was no missing data and all data from the 500 participants were analyzed in this study. Table 1 summarizes the socio-demographic characteristics of the women included in this study. The mean age of women was 48 years (standard deviation [SD] 5.5, range 40–69). Of the total, 18.0% had an education level of bachelor’s degree and above. The median monthly family income was Nepalese Rupee (NPR) 47,500 ranging from NPR 825 to NPR 298,397. Of the total, 44.8% were housewives. Around 20% of women had a family history of breast cancer. Of the total, 37.4% had family members from the health field (students or professionals). Only 15% of the women participated in the awareness program related to breast cancer.

**Table 1:** Socio-demographic characteristics of participants (n = 500).

Socio-demographic variables	n	%
Age* [mean (SD)]	48.2 (5.5)	
**Ethnicity**		
Brahmin/Chhetri	264	52.9
Janajati	220	44.0
Others (Dalit/Muslim/Madheshi)	16	3.2
**Religion**		
Hindu	466	93.2
Buddhism/Islam/Christianity	34	6.8
**Education**		
Illiterate	52	10.4
Can read and write only	83	16.6
Primary	58	11.6
Secondary	136	27.2
Higher secondary	81	16.2
Bachelor’s degree and above	90	18.0
**Occupation**		
Housewife	224	44.8
Business	208	41.6
Labor work	16	3.2
Services (Govt/private)	52	10.4
**Husband education (n = 475)**		
Illiterate	21	4.4
Can read and write only	22	4.6
Primary	43	9.1
Secondary	116	24.4
Higher secondary	88	61.1
Bachelor’s degree and above	185	38.9
**Husband’s occupation (n = 475)**		
Agriculture	24	5.1
Business	156	32.8
Labor work	42	8.8
Services (Govt/private)	168	35.4
Retried / foreign employment	85	17.9
**Monthly family Income (Median)** (1US$ = 110 NPR)		NPR 47,500
**Time taken to reach the nearest health facility**		
Less than 30 min	419	83.8
30 minutes and more	81	16.2
**Family history of breast cancer**		
Yes	104	20.8
No	396	79.2
**Participation in any breast cancer training/awareness program**		
Yes	73	14.6
No	427	85.4
**Family member from health field (student, health worker)**		
Yes	187	37.4
No	313	62.6

Table 2 shows a summary of the exposure variables. Among all variables, the mean score of perceived susceptibility towards breast cancer was the lowest with a score of 2.5 (SD 1.0).

**Table 2:** Summary table of exposure variables.

Exposure variables	Mean	SD
**Attitude**		
Mammography	3.7	0.5
Clinical breast examination	3.5	0.6
Breast self-examination	3.8	0.8
**Subjective norm**		
Mammography	3.4	0.7
Clinical breast examination	3.3	0.8
Breast self-examination	3.4	1.2
**Perceived behavioral control**		
Mammography	3.5	0.8
Clinical breast examination	3.5	0.6
Breast self-examination	2.8	1.4
**Risk perception**		
Perceived susceptibility	2.5	1.0
Perceived severity	3.6	1.0
Knowledge of breast cancer (Range: 0–21)	9.9	3.5
Breast cancer fatalism (Range: 0–11)	4.8	3.5

Table 3 illustrates the practice of breast cancer screening. Of the total, 3.4% of women had undergone MMG biennially, 7.2% had undergone CBE annually, and 14.4% practiced BSE monthly. Similarly, Table 4 shows the breast cancer screening intention. Around 20% of women expressed strong intention (strongly agree) to undergo BSE. More than half (64.6%) of women disagreed of having the intention to undergo MMG.

**Table 3:** Breast cancer screening behavior (N = 500).

Behavior of breast cancer screening	n	%
**MMG**		
Never	448	89.6
Occasionally	35	7.0
Biennially	17	3.4
**CBE**		
Never	400	80.0
Occasionally	64	12.8
Annually	36	7.2
**BSE**		
Never	293	58.6
Occasionally	135	27.0
Monthly	72	14.4

MMG: mammography, CBE: clinical breast examination, BSE: breast self-examination.

**Table 4:** Breast cancer screening intention (N = 500).

Intention to do breast cancer screening	Strongly Agree n (%)	Agree (%)	Neutral n (%)	Disagree n (%)	Strongly Disagree n (%)
MMG (biennially)	32 (6.4)	121 (24.2)	24 (4.8)	323 (64.6)	-
CBE (annually)	36 (7.2)	139 (27.8)	32 (6.4)	284 (56.8)	9 (1.8)
BSE (monthly)	99 (19.8)	235 (47.0)	80 (16.0)	72 (14.4)	14 (2.8)

MMG: mammography, CBE: clinical breast examination, BSE: breast self-examination.

Table 5 shows the result of the multivariable partial proportional odds model of the factors associated with breast cancer screening intention. After adjusting for confounders and other variables, women who participated in the awareness program of breast cancer were more likely to have the intention to undergo MMG (AOR = 2.69, 95% CI 1.42–5.11).

**Table 5:** Factors associated with breast cancer screening intention (N = 500).

Variables	MMG		CBE		BSE	
	AOR	(95% CI)	AOR	(95% CI)	AOR	(95% CI)
Participation in awareness programs						
No	1.00		1.00		1.00	
Yes	2.69 **	(1.42–5.11)	1.72	(0.92–3.19)	1.69	(0.75–3.85)
Fatalism	0.96	(0.88–1.05)	0.92 *	(0.86–0.99)	1.00	(0.93–1.08)
Susceptibility	1.06 *	(1.01–1.12)	1.08 **	(1.03–1.13)	1.04	(0.99–1.10)
Knowledge of breast cancer	1.01	(0.92–1.12)	1.04	(0.96–1.14)	0.96 ^a^	(0.88–1.05)
					1.18 ^b^ ***	(1.08–1.29)
					0.86 ^c^	(0.72–1.02)
Attitude towards behaviour	1.40 ***	(1.19–1.65)	1.25 ***	(1.11–1.41)	2.23^a^ ***	(1.67–2.97)
					2.91^b^ ***	(2.13–3.99)
					5.51^c^ ***	(2.04–14.86)
Subjective norms	2.18 ***	(1.81–2.62)	1.64 ***	(1.43–1.89)	1.44 ^a^	(0.98–2.14)
					1.68 ^b^ **	(1.19–2.37)
					13.13 ^c^ ***	(5.79–29.79)
Perceived behaviour control	1.96 ***	(1.65–2.34)	1.47 ^a^ ***	(1.19–1.81)	2.11 ***	(1.69–2.62)
			1.55 ^b^ ***	(1.24–1.95)		
			4.66 ^c^ ***	(3.10–7.01)		

*p<0.05,

**p<0.01,

***p<0.001 (Adjusted for age, no of children, education, occupation, family income, family members from a health background, time required to reach a nearby health facility, and family history of breast cancer) AOR: adjusted odds ratio, CI: confidence interval, MMG: mammography, CBE: clinical breast examination, BSE: breast self-examination. Dependent variable coding: SD&D = strongly disagree and disagree, N = neutral, A = agree, SA = strongly agree.

[Note: Only one set of AOR is presented for explanatory variables that meet the proportional odds assumption. For variables with non-proportional odds, three AORs are presented as symbolized by ’a’, ’b’, and ’c’ where, a = AOR for SD&D versus N, A and SA; b = AOR for SD&D or N versus A and SA; c = AOR for SD&D or N or A versus SA.]

Women who perceived themselves susceptible to breast cancer were more likely to have the intention to undergo MMG (AOR = 1.06, 95% CI 1.01–1.12) and CBE (AOR = 1.08, 95% CI 1.03–1.13). In contrast, women with high fatalistic beliefs were less likely to have the intention to undergo CBE (AOR = 0.92, 95% CI 0.86–0.99).

For MMG, women with a positive attitude towards MMG (AOR = 1.40, 95% CI 1.19–1.65), higher subjective norms (AOR = 2.18, 95% CI 1.81–2.62), and high perceived behavioral control (AOR = 1.96, 95% CI 1.65–2.34) were more likely to have the intention to undergo MMG.

Similarly, women who had positive attitudes towards CBE (AOR = 1.25, 95% CI 1.11–1.41) and had higher subjective norms (AOR = 1.64, 95% CI 1.43–1.89) were more likely to have the intention to undergo CBE. Having a higher perceived behavior control was associated with the likelihood of being in a higher agreement level (A and SA) to undergo CBE as opposed to being at neutral or below neutral level (AOR = 1.55, 95% CI 1.24–1.95). The effects became much stronger with increment in perceived behavior control, further, the largest effect was identified among the final level (i.e. SA versus A, N or SD&D).

In the case of BSE, women with higher attitude (AOR = 2.91, 95% CI 2.13–3.99), and those with better subjective norms (AOR = 1.68, 95% CI 1.19–2.37) were positively associated with increased odds of expressing higher agreement (A and SA) to undergo BSE rather than expressing neutral or disagreement. Women who had high perceived behavioral control were more likely to have intention to undergo BSE (AOR = 2.11, 95% CI 1.69–2.62). Findings revealed that having better knowledge of breast cancer increased the odds of a woman expressing higher agreement to undergo BSE rather than expressing neutral or disagreement (A and SA versus N, D&SD; AOR = 1.18, 95% CI 1.08–1.29), though it failed to achieve statistical significance in other categories.

## Discussion

All three components of TPB (Attitude, subjective norms, and perceived behavior control) were positively associated with the intention to undergo MMG, CBE, and BSE. Similarly, women who perceived themselves susceptible to breast cancer were more likely to have the intention to undergo MMG and CBE. Women who participated in the breast cancer awareness program were more likely to have the intention to undergo MMG. In contrast, women with fatalistic beliefs were less likely to have the intention to undergo CBE. Furthermore, with an increase in knowledge of breast cancer, women were more likely to have the intention to undergo BSE.

Our study findings align with the previous studies that have used TPB to predict different screening and healthy behavior intentions [45–47]. In our study, women were more likely to have the intention to undergo screening tests when they had a positive attitude towards a particular test. Positive attitude was found as an important factor to change behavior like adopting a healthy lifestyle [48]. People intend and are motivated to do such behavior which they believe can lead to positive outcomes [49]. Furthermore, in our study, women receiving screening suggestions from their family members and close ones were more likely to have intention to do it. People usually adhere to the advice given by trusted family members and close ones [50]. Social ties were found to have a positive influence in bringing healthy changes among participants in different settings [51–53]. Therefore, educating a person alone is not sufficient; the involvement of friends and family (social network) is also salient to effectively promote screening intention. It is particularly important for countries like Nepal, where women need permission from their husbands and in-laws before making any personal decision or health choices [54–56].

Likewise, screening intention was higher among women who believed in their capability to go for screening and perceived fewer barriers to screening. Similar to our finding, in a study conducted among Latina women in the United States, women expressed greater intention of receiving cervical cancer screening tests when they had high perceived behavior control [57].

It is worth considering particularly in low and middle-income countries like Nepal where screening services are not easily available. Many women are not financially competent to afford expensive services like MMG. Women should be involved in income-generating activities where they could support their expenses. Besides, a screening test like BSE can be done by a woman herself under complete privacy and autonomy. Proper training and awareness must be provided so that women perceived control to undergo those screenings which are free of cost and can be done independently. Evidently, in our study women who had high knowledge of breast cancer were more likely to have the intention to undergo BSE.

Another factor associated with the intention to undergo screening was perceived susceptibility. Women were more likely to have the intention to undergo MMG and CBE when they perceived themselves susceptibility to breast cancer. This is also an important factor to be considered particularly for low and middle-income countries like Nepal where people seek services only when they perceive themselves susceptible to health risks [58, 59]. Women did not perceive the need of undergoing screening until they had recognizable symptoms of breast cancer [60]. As a result, victims end up facing multiple challenges associated with late presentation, treatment difficulties, financial cost, etc. It is pivotal to make people realize that anyone can suffer from breast cancer and their timely screening practice can prevent them from other detrimental consequences. Conducting an awareness program is therefore key to promote screening behavior such as MMG which needs a more conscious decision. Evidently in our study, women who had participated in the awareness program were more likely to have the intention to undergo MMG. In our study intention to undergo BSE was not associated with perceived susceptibility. This could be because people usually prefer to seek help from health professionals when they actually perceived some kind of health threat.

Like perceived susceptibility, fatalism is a potential barrier preventing people from participation in health-promoting behaviors [61]. However, in our study, the fatalistic belief was associated with the intention to undergo CBE only. Women with high fatalistic beliefs were less likely to have the intention to undergo CBE. Previous studies have also presented the mixed result of fatalism in screening intentions [62, 63]. Therefore, there is a need for an in-depth exploration of this belief before making any concrete conclusion. Nonetheless one of the reasons for our findings could be because women in a religious country like Nepal are usually reluctant to show their private body parts to anyone including health professionals unless it is very essential. Unlike BSE (which is self-examination) and MMG (test conducted using a device), CBE is the manual palpation done by the health care professionals. Considering the nature of the test, women with religious and fatalistic beliefs might be reluctant to pursue it [64, 65]. Therefore, deep traditional and fatalistic beliefs should not be ignored while designing screening interventions.

This study has several limitations. First, women might have over-reported their intention to undergo and behavior of screening to avoid awkwardness due to further questioning on their intention and behavior. However, the chance of over-reporting is equal for all participants, so the association between the exposure variables and the outcome variables should not be affected by this bias. The study was conducted in the urban areas of Nepal. Therefore, it cannot be generalized to the entire population. However, urban areas were selected considering the availability and accessibility of the screening tests. Finally, women who did not agree to participate in this study might have a different attitude, fatalistic belief, and knowledge level, which is not reflected in this study.

Despite these limitations, this study provides a comprehensive understanding of different factors associated with breast cancer screening intention. This could be helpful to develop a culturally sensitive intervention to promote breast cancer screening in resource-limited settings. This study also provides information on breast cancer screening behaviors, knowledge level, and fatalistic beliefs of women towards breast cancer. Findings can pave a way for future studies on breast cancer screening behavior.

## Conclusion

This study revealed poor screening behavior of women living in Kathmandu Valley, Nepal. Findings highlight the importance of TPB (positive attitude, subjective norms, perceived behavior control) along with perceived susceptibility and fatalism in increasing intention to undergo screening tests. Thus, educating only an individual is not enough, the inclusion of family members and addressing deep fatalistic beliefs are crucial for the successful promotion of screening. Women should be encouraged to undergo screening timely even before the appearance of symptoms. Meanwhile, screening tests should be made available and approachable before advocating for those services. Most importantly, practical training on BSE should be provided so that women feel competent to carry out themselves. To conclude, multidimensional culturally sensitive interventions are necessary to promote breast cancer screening in Nepal.

## Supporting information

S1 Checklist STROBE checklist. (DOC)

## References

[1] Bray F, Ferlay J, Soerjomataram I, Siegel RL, Torre LA, Jemal A. Global cancer statistics 2018: GLOBOCAN estimates of incidence and mortality worldwide for 36 cancers in 185 countries. CA Cancer J Clin. 2018;68(6):394–424. 10.3322/caac.21492 30207593

[2] De Sanjose S, Tsu VD. Prevention of cervical and breast cancer mortality in low- and middle-income countries: a window of opportunity. Int J Womens Health. 2019;11:381–6. 10.2147/IJWH.S197115 31308762 PMC6617716

[3] Tfayli A, Temraz S, Abou R, Shamseddine A. Breast cancer in low- and middle-income countries: an emerging and challenging epidemic. J Oncol. 2010;2010:490631 10.1155/2010/490631 21209708 PMC3010663

[4] Yip CH, Taib NA. Challenges in the management of breast cancer in low- and middle-income countries. Futur Oncol. 2012;8(12):1575–83. 10.2217/fon.12.141 23231519

[5] American Cancer Society. Breast cancer early detection and diagnosis [Internet]. 2017 [cited 2018 Jan 31]. https://www.cancer.org/cancer/breast-cancer/screening-testsand-early-detection.html

[6] Anderson BO, Yip C-H, Smith RA, Shyyan R, Sener SF, Eniu A, et al Guideline implementation for breast healthcare in low-income and middle-income countries. Cancer. 2008;113(S8):2221–43. 10.1002/cncr.23844 18816619

[7] Provencher L, Hogue JC, Desbiens C, Poirier B, Poirier E, Boudreau D, et al Is clinical breast examination important for breast cancer detection? Curr Oncol. 2016;23(4):332–9. 27536182 10.3747/co.23.2881 PMC4974039

[8] Tara S, Agrawal CS, Agrawal A. Validating breast self-examination as screening modalities for breast cancer in eastern region of Nepal: a population-based study. Kathmandu Univ Med J. 2008;6(1):89–93. 18604121

[9] Blumen H, Fitch K, Polkus V. Comparison of treatment costs for breast cancer, by tumor stage and type of service. Am Heal Drug Benefits. 2016;9(1):23–32. 27066193 PMC4822976

[10] Poudel KK, Huang Z, Neupane PR, Steel R, Poudel JK. Hospital-based cancer incidence in Nepal from 2010 to 2013. Nepal J Epidemiol. 2017;7(1):659–65. 10.3126/nje.v7i1.17759 28970948 PMC5607447

[11] Fajdic J, Djurovic D, Gotovac N, Hrgovic Z. Criteria and procedures for breast-conserving surgery. Acta Inform Med. 2013;21(1):16–9. 23572855 10.5455/AIM.2013.21.16-19 PMC3610577

[12] Singh YP, Sayami P. Management of breast cancer in Nepal. J Nepal Med Assoc. 2009;48(175):252–7. 20795469

[13] Tfayli A, Temraz S, Abou Mrad R, Shamseddine A. Breast cancer in low- and middle-income countries: an emerging and challenging epidemic. J Oncol.2010; 2010:490631 10.1155/2010/490631 21209708 PMC3010663

[14] Rivera-Franco MM, Leon-Rodriguez E. Delays in breast cancer detection and treatment in developing countries. Breast Cancer. 2018;12:1178223417752677 10.1177/1178223417752677 29434475 PMC5802601

[15] Acharya SC, Jha AK, Manandhar T. Clinical profile of patients presenting with breast cancer in Nepal. Kathmandu Univ Med J. 2012;39(3):3–7. 10.3126/kumj.v10i3.8009 23434953

[16] Action Study Group. Health-related quality of life and psychological distress among cancer survivors in Southeast Asia: results from a longitudinal study in eight low- and middle-income countries. BMC Med. 2017;15(1):10 10.1186/s12916-016-0768-2 28081724 PMC5234136

[17] Youlden DR, Cramb SM, Dunn NA, Muller JM, Pyke CM, Baade PD. The descriptive epidemiology of female breast cancer: an international comparison of screening, incidence, survival, and mortality. Cancer Epidemiol. 2012;36(3):237–48. 10.1016/j.canep.2012.02.007 22459198

[18] Heidari M, Ghodusi M. The relationship between body esteem and hope and mental health in breast cancer patients after mastectomy. Indian J Palliat Care. 2015;21(2):198–202. 10.4103/0973-1075.156500 26009674 PMC4441182

[19] Latalski M, Kulik TB, Skórzyńska H, Zołnierczuk D. Social consequences of breast cancer in women suffering from the disease. Wiad Lek. 2001;54(7–8):391–8. 11641894

[20] Pandey A, Rao N. Treatment-seeking behavior and cost of care among cancer patients in Nepal. J Fam Med Community Health. 2015;2(1):1024.

[21] Alexandraki I, Mooradian AD. Barriers related to mammography use for breast cancer screening among minority women. J Natl Med Assoc. 2010;102(3):206–18. 10.1016/s0027-9684(15)30527-7 20355350

[22] Patel K, Kanu M, Liu J, Bond B, Brown E, Williams E, et al Factors influencing breast cancer screening in low-income African Americans in Tennessee. J Community Health. 2014;39(5):943–50. 10.1007/s10900-014-9834-x 24554393 PMC4165808

[23] Griva F, Anagnostopoulos F, Madoglou S. Mammography screening and the Theory of Planned Behavior: suggestions toward an extended model of prediction. Women Health. 2010;49(8):662–81. 10.1080/03630240903496010 20183107

[24] Pant PR, Dangol D. Kathmandu Valley profile briefing paper. [Internet].2009. [cited 2018 Oct 27];1–7 https://www.eastwestcenter.org/fileadmin/resources/seminars/Urbanization_Seminar/Kathmandu_Valley_Brief_for_EWC___KMC_Workshop__Feb_2009_.pdf

[25] Oldach BR, Katz ML. Health literacy and cancer screening: a systematic review. Patient Educ Couns. 2014;94(2):149–57. 10.1016/j.pec.2013.10.001 24207115 PMC3946869

[26] Godin G, Gagné C, Maziade J, Moreault L, Beaulieu D, Morel S. Breast cancer: The intention to have a mammography and a clinical breast examination—application of the theory of planned behavior. Psychol Health. 2001;16(4):423–41.

[27] Mayo R, Hunter A. Fatalism toward breast cancer among the women of Ghana. Health Care Women Int. 2003;24(7):608–16. 10.1080/07399330390217752 14627208

[28] Satyal K. Cervical cancer screening behavior among Nepalese women. PhD [dissertation]. USA:George Mason University;2013.

[29] Bhandari PM, Thapa K, Dhakal S, Bhochhibhoya S, Deuja R, Acharya P, et al Breast cancer literacy among higher secondary students: results from a cross-sectional study in western Nepal. BMC Cancer. 2016;16(1):119 10.1186/s12885-016-2166-8 26887650 PMC4758038

[30] Ajzen I. The theory of planned behavior. Organ Behav Hum Decis Process. 1991;50(2):179–211.

[31] Espinosa de los Monteros K, Gallo LC. The relevance of fatalism in the study of Latinas’ cancer screening behavior: A systematic review of the literature. Int J Behav Med. 2011;18(4):310–8. 10.1007/s12529-010-9119-4 20953916 PMC3212691

[32] Kim SE, Pérez-Stable EJ, Wong S, Gregorich S, Sawaya GF, Walsh JME, et al Association between cancer risk perception and screening behavior among diverse women. Arch Intern Med. 2008;168(7):728–34. 10.1001/archinte.168.7.728 18413555 PMC4031902

[33] Nahidi F, Dolatian M, Roozbeh N, Asadi Z, Shakeri N. Effect of health-belief-model-based training on performance of women in breast self-examination. Electron physician. 2017;9(6):4577–83. 10.19082/4577 28848633 PMC5557138

[34] Day AK, Wilson CJ, Hutchinson AD, Roberts RM. The role of skin cancer knowledge in sun-related behaviours: A systematic review. J Health Psychol. 2014;19(9):1143–62. 10.1177/1359105313485483 23682066

[35] Ramírez AS. Fatalism and cancer risk knowledge among a sample of highly acculturated Latinas. J Cancer Educ. 2014;29(1):50–5. 10.1007/s13187-013-0541-6 24078314 PMC5464787

[36] Ministry of Health and Population, New ERA, ICF International Inc. Nepal Demographic and Health Survey 2016. [Internet]. Kathmandu, Nepal: Ministry of Health; 2017 [cited 2018 Feb 21] https://dhsprogram.com/pubs/pdf/FR257/FR257[13April2012].pdf.

[37] Bynum JPW, Braunstein JB, Sharkey P, Haddad K, Wu AW. The influence of health status, age, and race on screening mammography in elderly women. Arch Intern Med. 2005;165(18):2083–8. 10.1001/archinte.165.18.2083 16216997

[38] Cullati S, Charvet-Bérard AI, Perneger TV. Cancer screening in a middle-aged general population: factors associated with practices and attitudes. BMC Public Health. 2009;9(1):118. 19402895 10.1186/1471-2458-9-118 PMC2685378

[39] Haber G, Ahmed NU, Pekovic V. Family history of cancer and its association with breast cancer risk perception and repeat mammography. Am J Public Health. 2012 12;102(12):2322–9. 10.2105/AJPH.2012.300786 23078489 PMC3519312

[40] Williams R. Generalized ordered logit/partial proportional odds models for ordinal dependent variables. Stata J. 2006;6(1):58–82.

[41] Brant R. Assessing Proportionality in the Proportional Odds Model for Ordinal Logistic Regression. Biometrics. 1990;46(4):1171 2085632

[42] Williams R. Understanding and interpreting generalized ordered logit models. J Math Sociol. 2016;40(1):7–20.

[43] Kuha J. AIC and BIC: Comparisons of assumptions and performance. Sociol Methods Res. 2004;33(2):188–229.

[44] IDRE. Regression with stata chapter 2: regression diagnostics. Institute for Digital Research and Education. University of California Los Angeles; 2015. [cited date 2018 Feb 21] http://www.ats.ucla.edu/stat/stata/webbooks/reg/chapter2/statareg2.htm.

[45] Huang J, Wang J, Pang TWY, et al Does theory of planned behaviour play a role in predicting uptake of colorectal cancer screening? A cross-sectional study in Hong Kong. BMJ Open. 2020;10(8):e037619 10.1136/bmjopen-2020-037619 32764087 PMC7412617

[46] Roncancio AM, Ward KK, Fernandez ME. Understanding cervical cancer screening intentions among latinas using an expanded theory of planned behavior model. Behav Med. 2013;39(3):66–72. 10.1080/08964289.2013.799452 23930898 PMC4895917

[47] Lin N, Roberts KR. Using the theory of planned behavior to predict food safety behavioral intention: A systematic review and meta-analysis. Int J Hosp Manag. 2020;90:102612.

[48] Rivera MM, Leon-Rodriguez E. Delays in breast cancer detection and treatment in developing countries. Breast Cancer Auckl. 2018;12:117. 10.1177/1178223417752677 PMC5802601 29434475

[49] Mäntyselkä P, Kautiainen H, Miettola J. Beliefs and attitudes towards lifestyle change and risks in primary care—A community-based study. BMC Public Health. 2019;19(1):1049 10.1186/s12889-019-7377-x 31382938 PMC6683394

[50] Kiyang LN, Labrecque M, Doualla-Bell F, Turcotte S, Farley C, Cionti Bas M, et al Family physicians’ intention to support women in making informed decisions about breast cancer screening with mammography: a cross-sectional survey. BMC Res Notes. 2015;8(1):663 10.1186/s13104-015-1608-8 26555569 PMC4640384

[51] Robinson CA, Trent M, Ellen JM, Matson PA. Rethinking Urban Female Adolescents’ Safety Net: The Role of Family, Peers, and Sexual Partners in Social Support. Am J Heal Promot. 2020;34(4):431–5. 10.1177/0890117119896194 PMC7166135 31867977

[52] Miller-Graff LE, Scheid CR, Guzmán DB, Grein K. Caregiver and family factors promoting child resilience in at-risk families living in Lima, Peru. Child Abus Negl. 2020;108:104639 10.1016/j.chiabu.2020.104639 32758713

[53] Hu J, Wu Y, Ji F, et al Peer Support as an Ideal Solution for Racial/Ethnic Disparities in Colorectal Cancer Screening. Dis Colon Rectum. 2020;63(6):850–8. 10.1097/DCR.0000000000001611 32167969

[54] Ohashi A, Higuchi M, Labeeb SA, et al Family support for women’s health-seeking behavior: a qualitative study in rural southern Egypt (Upper Egypt). Nagoya J Med Sci. 2014;76(1–2):17–25. 25129988 PMC4345735

[55] Allendorf K. The Quality of Family Relationships and Use of Maternal Health-care Services in India. Stud Fam Plann. 2010;41(4):263–76. 10.1111/j.1728-4465.2010.00252.x 21465727 PMC4845731

[56] Simkhada B, Porter MA, van Teijlingen ER. The role of mothers-in-law in antenatal care decision-making in Nepal: a qualitative study. BMC Pregnancy Childbirth. 2010;10(1):1–10. 10.1186/1471-2393-10-34 20594340 PMC2910658

[57] Roncancio AM, Ward KK, Sanchez IA, Cano MA, Byrd TL, Vernon SW, et al Using the Theory of Planned Behavior to Understand Cervical Cancer Screening among Latinas. Health Educ Behav. 2015;42(5):621–6. 10.1177/1090198115571364 25712240 PMC4932857

[58] Basharat S, Shaikh BT, Rashid HU, Rashid M. Health seeking behaviour, delayed presentation and its impact among oral cancer patients in Pakistan: A retrospective qualitative study. BMC Health Serv Res. 2019;19(1):715 10.1186/s12913-019-4521-3 31638970 PMC6805330

[59] Widayanti AW, Green JA, Heydon S, Norris P. Health-seeking behavior of people in Indonesia: A narrative review Vol. 10, Journal of Epidemiology and Global Health. Atlantis Press International; 2020 p. 6–15. 10.2991/jegh.k.200102.001 PMC7310809 32175705

[60] Lee S-Y, Lee EE. Cancer screening in Koreans: a focus group approach. BMC Public Health. 2018;18(1):254 10.1186/s12889-018-5147-9 29444640 PMC5813376

[61] Perfetti AR. Fate and the clinic: A multidisciplinary consideration of fatalism in health behavior. Med Humanit.2018;44(1):59–62. 10.1136/medhum-2017-011319 28993427

[62] Bakan AB, Aslan G, Yıldız M. Determination of Breast Cancer Fatalism in Women and the Investigation of the Relationship Between Women’s Cervical Cancer and Pap Smear Test Health Beliefs with Religious Orientation and Fatalism. J Relig Health. 2020;1–21. 2 33123972 10.1007/s10943-020-01108-2

[63] Abraído-Lanza AF, Martins MC, Shelton RC, Flórez KR. Breast Cancer Screening Among Dominican Latinas: A Closer Look at Fatalism and Other Social and Cultural Factors. Heal Educ Behav. 2015;42(5):633–41. 10.1177/1090198115580975 PMC5385102 25869406

[64] Attum B, Shamoon Z. Cultural Competence in the Care of Muslim Patients and their Families [Internet]. StatPearls. StatPearls Publishing; 2018 [cited 2020 Dec 2]. https://www.ncbi.nlm.nih.gov/books/NBK499933/29763108

[65] Lee SY. Cultural Factors Associated with Breast and Cervical Cancer Screening in Korean American Women in the US: An Integrative Literature Review Vol. 9, Asian Nursing Research. Korean Society of Nursing Science; 2015 p. 81–90. 10.1016/j.anr.2015.05.003 26160234

