# Supplementary material for: Factors associated with breast cancer screening intention in Kathmandu Valley, Nepal
Source: PLoS One. 2021 Jan 22;16(1):e0245856. doi: 10.1371/journal.pone.0245856 (PMC7822561; doi:10.1371/journal.pone.0245856)
Supplement: S1 Checklist — (DOC) [file pone.0245856.s001.doc]

STROBE Statement—Checklist of items that should be included in reports of ***cross-sectional studies***

|  | Item No | Recommendation | Page No. | Relevant text from the manuscript |
| --- | --- | --- | --- | --- |
| **Title and abstract** | 1 | (*a*) Indicate the study’s design with a commonly used term in the title or the abstract | 2 | Line 26; Methods subsection of Abstract: “A cross-sectional study...” |
|  |  | (*b*) Provide in the abstract an informative and balanced summary of what was done and what was found | 2-3 | Line 17-48 (Abstract Section) |
| Introduction | | |  |  |
| Background/rationale | 2 | Explain the scientific background and rationale for the investigation being reported | 4-5 | Line 64-98 (Introduction: “Breast cancer…”) |
| Objectives | 3 | State specific objectives, including any prespecified hypotheses | 5 | Line 96-98; Introduction section, last paragraph (“Therefore study aimed…”) |
| Methods | | |  |  |
| Study design | 4 | Present key elements of study design early in the paper | 5 | Line 101; “Settings and Participants” (A cross-sectional study…) |
| Setting | 5 | Describe the setting, locations, and relevant dates, including periods of recruitment, exposure, follow-up, and data collection | 5  9-10 | Line 101-108(Subsection: Settings and Participants)  Line 200-205 (Section: Data collection) |
| Participants | 6 | (*a*) Give the eligibility criteria, and the sources and methods of selection of participants | 5-6 | Line 109-111(Settings and Participants “…eligible participants...”) |
| Variables | 7 | Clearly define all outcomes, exposures, predictors, potential confounders, and effect modifiers. Give diagnostic criteria, if applicable | 6-9 | Line 132-199; subsection: Exposure variables and assessment, Outcome variables and assessment, Potential confounders and assessment |
| Data sources/ measurement | 8* | For each variable of interest, give sources of data and details of methods of assessment (measurement). Describe comparability of assessment methods if there is more than one group | 6-9 | Line 132-199; subsection: Exposure variables and assessment, Outcome variables and assessment, Potential confounders and assessment |
| Bias | 9 | Describe any efforts to address potential sources of bias | 6 | Line 125-130 (pre-testing and Cronbach alpha) |
| Study size | 10 | Explain how the study size was arrived at | 6 | Line 112-119; Subsection: Settings and Participants; “The sample size was determined…” |
| Quantitative variables | 11 | Explain how quantitative variables were handled in the analyses. If applicable, describe which groupings were chosen and why | 6-9 | Line 132-199; subsection: Exposure variables and assessment, Outcome variables and assessment, Potential confounders and assessment |
| Statistical methods | 12 | (*a*) Describe all statistical methods, including those used to control for confounding | 10 | Line 206-214; Section: Data analysis; “Simple and multiple logistic regression analyses were conducted...” |
|  |  | (*b*) Describe any methods used to examine subgroups and interactions |  | Not applicable |
|  |  | (*c*) Explain how missing data were addressed |  | Not applicable |
|  |  | (*d*) If applicable, describe analytical methods taking account of sampling strategy |  | Not applicable |
|  |  | (*e*) Describe any sensitivity analyses |  | Not applicable |
| Results | | |  |  |
| Participants | 13* | (a) Report numbers of individuals at each stage of study—eg numbers potentially eligible, examined for eligibility, confirmed eligible, included in the study, completing follow-up, and analysed | 10 | Line 224-225; Section: Result; “Out of 529 women who were approached….” |
|  |  | (b) Give reasons for non-participation at each stage |  |  |
|  |  | (c) Consider use of a flow diagram |  |  |
| Descriptive data | 14* | (a) Give characteristics of study participants (eg demographic, clinical, social) and information on exposures and potential confounders | 11-14 | Line 223-234; Table 1 Sociodemographic characteristics of participants;  Table 2 Summary table of exposure variables |
|  |  | (b) Indicate number of participants with missing data for each variable of interest |  |  |
| Outcome data | 15* | Report numbers of outcome events or summary measures | 13 | Line 236-239 |
| Main results | 16 | (*a*) Give unadjusted estimates and, if applicable, confounder-adjusted estimates and their precision (eg, 95% confidence interval). Make clear which confounders were adjusted for and why they were included | 17 | Line 273; Table 5 Factors associated with breast cancer screening intention |
|  |  | (*b*) Report category boundaries when continuous variables were categorized | 8-9 | Line 132-199; subsection: Exposure variables and assessment, Outcome variables and assessment, Potential confounders and assessment  “Median of the total score was taken as a cut-off point to categorize...” |
|  |  | (*c*) If relevant, consider translating estimates of relative risk into absolute risk for a meaningful time period |  | Not applicable |
| Other analyses | 17 | Report other analyses done—eg analyses of subgroups and interactions, and sensitivity analyses |  | Not applicable |
| Discussion | | |  |  |
| Key results | 18 | Summarise key results with reference to study objectives | 14-16 | Line 241-271; Section: Results |
| Limitations | 19 | Discuss limitations of the study, taking into account sources of potential bias or imprecision. Discuss both direction and magnitude of any potential bias | 18-19 | Line 312-320; Section: Discussion; “This study has several limitations…” |
| Interpretation | 20 | Give a cautious overall interpretation of results considering objectives, limitations, multiplicity of analyses, results from similar studies, and other relevant evidence | 17-19 | Line 278-326; Section: Discussion |
| Generalisability | 21 | Discuss the generalisability (external validity) of the study results | 19 | Line 316-326; Section: Discussion; “...be generalized to the entire population…” |
| Other information | | |  |  |
| Funding | 22 | Give the source of funding and the role of the funders for the present study and, if applicable, for the original study on which the present article is based |  | Not applicable |

*Give information separately for exposed and unexposed groups.

**Note:** An Explanation and Elaboration article discusses each checklist item and gives methodological background and published examples of transparent reporting. The STROBE checklist is best used in conjunction with this article (freely available on the Web sites of PLoS Medicine at http://www.plosmedicine.org/, Annals of Internal Medicine at http://www.annals.org/, and Epidemiology at http://www.epidem.com/). Information on the STROBE Initiative is available at www.strobe-statement.org.
