# Supplementary material for: Factors associated with breast cancer screening intention in Kathmandu Valley, Nepal
Source: PLoS One. 2021 Jan 22;16(1):e0245856. doi: 10.1371/journal.pone.0245856 (PMC7822561; doi:10.1371/journal.pone.0245856)
Supplement: Attachment — Submitted filename: Response to Reviewers.docx [file pone.0245856.s002.docx]

Dear Editor and Reviewer,

We highly appreciate your meticulous review and constructive comments and suggestion. Please find below our response to your questions and comments. We have revised our manuscript incorporating your comments and suggestions.

**Comments from Editor**

*1. Please ensure that your manuscript meets PLOS ONE's style requirements, including those for file naming. The PLOS ONE style templates can be found at*

[*https://journals.plos.org/plosone/s/file?id=wjVg/PLOSOne_formatting_sample_main_body.pdf*](about:blank) *and*

[*https://journals.plos.org/plosone/s/file?id=ba62/PLOSOne_formatting_sample_title_authors_affiliations.pdf*](about:blank)

**Author Response:** We have duly read through the PLOS ONE’s style requirements and provided template files. We would like to ensure that our manuscript meets PLOS ONE’s style requirements, including file naming.

*2. Please address the following:*

*a) Please include additional information regarding the survey or questionnaire used in the study and ensure that you have provided sufficient details that others could replicate the analyses. For instance, if you developed a questionnaire as part of this study and it is not under copyright more restrictive than CC-BY, please include a copy, in both the original language and English, as Supporting Information. Besides, please provide details as to where the women were recruited from who participated in the pre-testing of this tool.*

**Author Response:** The detailed information regarding the survey or questionnaire used in the study has been provided in the “survey tools” and “variable” sub-section of the manuscript. We would also like to ensure that we have provided sufficient details so that others could replicate the analyses. Also, we would like to clarify that new questionnaires were not developed in this study, instead only the Nepali translation of the original English/French questionnaires was done in this study. Formal permissions were received from the original authors of the questionnaires via email before using their questionnaires in this study. We have properly cited and credited the original developers of the questionnaires throughout our manuscript.

Regarding the recruitment of participants for pretest, a total of 50 Nepalese women were included (around 10% of the target sample size). The pretest was conducted among women meeting the inclusion criteria and living in ward number 32 of Kathmandu, Nepal. These details have been added in the revised manuscript. Please refer to line number 125-127 in “Survey tools” sub-section.

*b) Please state whether or not the "expert panel" provided written, informed consent for their inclusion in this study concerning questionnaire development*.

**Author Response:** For the validation of the translated Nepali questionnaire, we performed three necessary steps as suggested by the WHO guidelines which include – 1) forward-translation and 2) back-translation by an independent translator and 3) review by an expert panel. The expert panel was informed about the study objectives and was approached for their voluntary participation to review the translated questionnaire. They were also informed about the non- traceability of their work and ability to withdraw from the process anytime without any clarification. All expert panel members agreed to review the translated questionnaire for clarity of expression, appropriateness of questionnaires for the target population and inconsistencies in languages. Discussions and meetings were organized with the experts to receive their suggestions and comments. Apart from the verbal agreement, any written informed consent for their voluntary participation was not obtained and was deemed not to be necessary. In the acknowledgement section of the manuscript, we have already expressed our sincere thanks to the expert panel and translators who helped in translation and evaluation of translated questionnaire. Please refer to line number 339-340 in the “Acknowledgements” section.

*c) Please refer to any sample size calculations performed before participant recruitment. If these were not performed please justify the reasons. Please refer to our statistical reporting guidelines for assistance (*[*https://journals.plos.org/plosone/s/submission-guidelines.#loc-statistical-reporting*](about:blank#loc-statistical-reporting)*).*

**Author Response**: The details on sample size calculation has been added in the revised manuscript. Please refer to line number 112-119 in the “Methods” section.

The sample size was determined using the OpenEpi sample size calculator for a cross-sectional study with a power of 80%, and a confidence level of 95%. As reported in a previous study with similar study design (Oldbach et al, 2014), we assumed that 7.1% of people with good literacy and 1.8% of people with low literacy practiced breast cancer screening.

The calculated minimum required sample size was 476. Considering 10% dropout, the target sample size was increased to 529. Compare to other factors included in this study, literacy required a larger sample size to show association with the independent variable, so it was considered for calculating the sample size.

**Reviewers' comments:**

*Reviewer #1: Dear Author(s),
This manuscript is overall reasonable. Your look on this issue is appreciable and your effort is admirable, short of a few points recommended in below:*

Dear Reviewer,

We highly appreciate your meticulous review and constructive comments and suggestion. Please find below our response to your comments. We have revised our manuscript by incorporating your comments and suggestions.

*1. The number of keywords is high. Please apply the MeSH part on* [*pubmed.com*](about:blank) *and extract the most related keywords.*

**Author Response**: We have revised the keywords using the online “MeSH on Demand” tool.

Revised Keywords: Breast cancer screening, Intention, Mammography, Breast self-examination, Nepal. Initially there were nine keywords, now we have reduced to five.

*2. Please specify and account for type of sampling obviously. As well, the grounds of why choosing this kind of sampling. Why are not cluster sampling opted?*

**Author Response**: Proportionate random sampling was used as a sampling method in this study. Please refer to line number 102-108.

As suggested, cluster sampling would have been an easier and economical method, however, random sampling was opted for better representativeness of the samples (greater precision).

*3. In the method part, 3 outcomes were numbered and after that, it was mentioned using multiple and logistic regression models. If there are 3 outcomes (responses), multivariate models should be applied, otherwise, 3 outcomes should enter in each model one at the time or 3 items together comprise one item (outcome or response). I know what you mean, however, I anticipate you to edit this part of abstract.*

**Author Response**: As per suggestion, we have rephrased the sentence to clarify the analysis approach in the abstract as follows: “The analysis was conducted separately for each outcome variable using multiple logistic regression models”.

*4. In Method part-Survey tools, please report Cronbach’s alpha (structured questionnaire).*

**Author Response**: We have added the value of Cronbach’s alpha under the “survey tools” sub-section in the “method” part. Cronbach's alphas for each construct (or subscale or related items) has been explained in the “variable” sub-section. Please refer to line 127-130.

*5. In Outcome variable and assessment- Intention to have breast cancer screening part, why was this variable transformed to binary outcome? You will lose the main information with this action. Please do not change this variable and use ordinal logistic regression model unless there is a valid study which previously indicated you can transform this outcome likewise Potential confounders and assessment- Knowledge of breast cancer. Please do not transform the ordinal variables to binary variables. This could reduce the validity of the results of analysis.*

**Author Response:** We agree that the ordinal outcome variable should have been analyzed with ordinal logistic regression for higher statistical power. However, we dichotomized the outcome variables and employed binary logistic regression because of the following reasons:

1) The questionnaire adopted in this study is a validated questionnaire developed by *Godin et al* *2001[ref 1 below].* In their paper, *Godin et al* have also later on dichotomized the intention outcome variable into two categories during their analysis. Similarly, another study by *Hart et al 2009 [ref 2 below]* has also dichotomized the screening intention outcome variable into two categories and applied binary logistic regression.

2) Moreover, as per our literature review of the studies using TPB, behaviors and intentions are usually defined and measured dichotomously. Every behavior outcomes are measured as performing or not performing the behavior. Similarly, intentions are measured as having or not having an intention. Referring to the previous research in our field, we also aimed to evaluate for two categories of intentions instead of evaluating five categories.

*[ref 1]. Godin G, Gagné C, Maziade J, Moreault L, Beaulieu D, Morel S. Breast cancer: The intention to have a mammography and a clinical breast examination-application of the theory of planned behavior. Psychology and Health. 2001 Jul 1;16(4):423-41.*

*[ref 2]. Hart SL, Bowen DJ. Sexual orientation and intentions to obtain breast cancer screening. Journal of Women's Health. 2009 Feb 1;18(2):177-85.*

*6. I did not understand what you mean? ” Additional support was also provided by political leaders and community workers of the respective areas” please omit this part.*

**Author Response**: We have removed this sentence from the manuscript.

*7. Please transmit Ethics and Data analysis part before the introduction of your variables and avoid many subtitles for Method parts. This can cause misleading.*

**Author Response:** We have minimized the subtitles for the “Methods” section in the revised manuscript. Please note that referring to the format of recently published cross-sectional studies in PLOS ONE journals, we have kept the “Ethics” and “Data analysis” part just before the “Results” section as it was before.

*8. Is there any test to display outlier or influential data? Please report. Moreover please check the pre-assumptions of logistic models.*

**Author Response**: Identification of potential outlier or influential data were checked using three diagnostic statistic – 1) Standardized Pearson residual, 2) deviance residual, and 3) Pregibon leverage (hat diagonal). These diagnostic tests were performed in STATA software. We noticed one outlier for the MMG model (ID: 117), two for CBE (ID: 361,372), and three for BSE (ID: 221, 237, 443). However, these detected observations have had less leverage during the Pregibon Leverage check. Hence it was concluded that none of these observations made any significant impact on logistic regression results within the context of a total sample size of 500. Therefore, all observations remained in the final analysis. Please refer to line 212-214 in “Data analysis” section.

We would like to ensure that all the pre-assumptions of logistic models were also checked and confirmed.

*9. The categories of patients’ educational level could be misleading. Higher secondary and above is not a reasonable cut off for this category. Either this variable should be retained continuously (with the years of education) or divided into more categories to find clear results.*

**Author Response:** As per your suggestion, the categories of participants' education levels have been divided into more categories. Please refer to the revised version of the manuscript. The old category of “higher secondary and above” has been divided again into three categories “higher secondary”, “bachelor level” and “master level and above”. However, in the survey only five women had a master's degree and above, so the “master’s degree and above” category was merged with the “bachelor level” category. All the analyses were updated again employing these new categories. We did not find any change in the relationship between independent and dependent variables. Please refer to the updated Table 5 in line 273.

*10. Please state why the median criterion reported for Monthly Income covariate.*

**Author Response**: As mentioned in the manuscript in line number 228, the monthly family income of the participants was diverse. Therefore, the median was used to properly represent the sample characteristics in descriptive statistics. However, while conducting regression analysis, monthly income was treated as a continuous variable.

*11. Please interpret the results using the odds ratio and the coefficients affected significantly the responses.*

**Author Response**: As suggested, we have presented the result of logistic regression using odds ratios(adjusted), confidence interval, and p-value in Table 5. Similarly, in the narrative also, the results have been interpreted using the adjusted odds ratios. The significantly associated factors have been clearly identified using the “*” symbol in Table 5. Please refer to the “Results” section of the manuscript.

*12. There is not any path diagram or (DAG) and figure to illustrate the results. Please confirm the results with figures and diagrams.*

**Author Response**: In our cross-sectional design setting, we mainly aimed for statistical association study rather than causal inference or path analysis. We, therefore, would like to be cautious and avoid presenting any causal diagrams such as DAGs without substantial evidence of a true causal effect. Furthermore, in scenarios containing a large number of variables, DAGs become multifaceted, complex, and less informative. However, under the Methods section, we have thoroughly explained all the independent variables, covariates/confounders, and outcome variables. All variables were selected based on prior knowledge, evidence, and theories as cited throughout the manuscript.
