# Supplementary material for: Factors associated with breast cancer screening intention in Kathmandu Valley, Nepal
Source: PLoS One. 2021 Jan 22;16(1):e0245856. doi: 10.1371/journal.pone.0245856 (PMC7822561; doi:10.1371/journal.pone.0245856)
Supplement: Attachment — Submitted filename: Response to Reviewer_20201216.docx [file pone.0245856.s004.docx]

**Response to Reviewer and Editor comments**

**Editor’s latest comment:** Thanks for your revision. However, I and the reviewer believe that you need to reanalyze the data. Therefore, you have the last chance to improve the manuscript. Unfortunately, I will reject it if you submit the manuscript without further analyzing.

Please understand that it is crucial to address reviewer’s comments or provide a strong justification for not revising.

**Reviewer’s latest comment:** I would like you either re-analyze your work or present the valid evidence to support your statistical analysis. Unfortunately, your second edition does not have to meet the Plos One criteria too and this article did not reach the compulsory merits. I am just able to channel your statistical inference to further research and learning more about using the categorical and dichotomize analysis precisely. It could be useful for future research: https://www.ncbi.nlm.nih.gov/pmc/articles/PMC1977443/pdf/brjcancer00075-0183.pdf

**Reviewer’s past comments related to above**: In Outcome variable and assessment- Intention to have breast cancer screening part, why was this variable transformed to binary outcome? You will lose the main information with this action. Please do not change this variable and use ordinal logistic regression model unless there is a valid study which previously indicated you can transform this outcome likewise Potential confounders and assessment- Knowledge of breast cancer. Please do not transform the ordinal variables to binary variables. This could reduce the validity of the results of analysis and please check the pre-assumptions of logistic models.

**Author Response:** Thank you for your detailed review, constructive comments, and suggestion. Concurring with reviewer/editor’s comments, we have reanalyzed the data using ordinal regression. Accordingly, we have revised/updated all affected result tables and narratives of our manuscript. Please kindly refer to “*Manuscript with track changes.docx*” to easily locate the changes made throughout the manuscript. A general overview of the changes made are described below:

1) In our earlier submitted version of the manuscript, we had transformed the ordinal outcome variable into a binary outcome and employed binary logistic regression. However, in this revised version, we have reanalyzed the data using an ordinal regression model concurring with the reviewer’s suggestion. Please kindly refer to the “*Data analysis”* section of the revised manuscript (lines ‘205-245’).
2) In the earlier submitted version of the manuscript, we had dichotomized the ‘knowledge’ and ‘fatalism’ variables as high/low level. However, in this revised version, we have used those variables as ‘continuous variables’ concurring with the reviewer’s suggestion. Please kindly refer to lines ‘153-155,’ and ‘184-185’ of the revised manuscript and also the updated tables- “Table 2” and “Table 5”.

*Extracted changes from the revised manuscript for your convenience:*

*Line 153-155: “…. The total score for fatalism (range 0-11) was calculated by adding responses of all 11 items which were coded as yes=1 and no=0. Fatalism was treated as a continuous variable and its’ higher value represented higher fatalism. ….”*

*Line 184-185: “… The total score for knowledge was calculated by adding scores of all 21 items and it was treated as a continuous variable… .”*

*Line 205-245: “… . Due to the ordinal nature of outcome variables, data were first fitted with the standard ordinal logistic regression (i.e. proportional odds model (POM)) model. However, POM was found inappropriate due to the violation of a proportional odds assumption for some independent variables. The proportional odds assumption was checked using a series of Wald tests, and Brant tests [40, 41]. The other alternatives were PPOM and fully unconstrained generalized ordered logit model (GOLM). PPOM relaxes the proportional odds assumptions for only those variables where it is violated. Whereas, GOLM relaxes the assumption for all variables, even if the assumption was violated by a few of them [42], resulting in too many parameters. Therefore, PPOM is usually considered a more efficient alternative to the GOLM [42]. Nonetheless, both PPOM and GOLM models were compared based on the Akaike Information Criterion (AIC) and Bayes’ Information Criterion (BIC) [43], and the PPOM model was found to have smaller AIC and BIC statistics. We, therefore, used PPOM as the final model to assess the factors associated with the intention to undergo screening. ..”*
